# Correspondence

**Published:** 1864-01

**Authors:** 


### CORRESPONDENCE.

Editor Medical and Surgical Journal:

*Dear Sir:*—It is with much regret that I feel obliged to intrude a second time upon the patience of your readers, but the style and tenor of Dr. Peters' article in your last issue in reply to what "Medicus" said in the November number, is so peculiar, that I cannot pass it over without a slight notice.

Plato says that "In writing we ought to hide ourselves, to disappear, to make the world forget us, that we may present nothing but the truths we wish to impress." I was forcibly reminded of this saying of Plato, when reading the Doctor's response to "Medicus" on "Spontaneous Salivation." The intense desire to get before the public in some way induces many a young man to follow a course precisely contrary to that advised above. It is no unusual thing consequently, to find ourselves delving deep in chaff in a vain search for a kernel of wheat. "Medicus" professes no taste for "trashy literature." but prefers plain common sense teaching in all things. But in thought, word and style, "*suus quisque mos sum.*" Nor does "Medicus" like that vain display of erudition, lavished without choice and without taste, nor that pomp of words which have no meaning.

Now I think I have satisfactorily explained our difference of "literary" ideas, and will proceed. With the "Medical Staff of the Army as a body," I have found no fault and introduced no *slur.* Perhaps Dr. Peters would like to enlist the whole Medical Army Staff in a body for the purpose of obliterating "Medicus." I deny making a charge or casting a slur upon them as a body, but individually. "Medicus" knows whereof he speaks, and further knows cases to which his memory cannot revert without a shudder. Some of them it is true were not witnessed, others were. At the risk of being tedious I will dare the patience of your readers and relate a case or two merely to show why I said what I did, and to prove to those who have not seen for themselves that my remarks were true, and that individuals for whom I intended them cannot escape obloquy should they follow Dr. P.'s example and invite the whole Medical Staff of the Army to their assistance. A sick officer from the 21st Regiment arrives at a hotel in Washington and promptly sends for the surgeon in charge of the district, who visits the patient, gives him a severe reprimand for leaving his regiment; attributes it to cowardice, thus adding insult to injury, and leaves to repeat his visit in two or three days, each time asserting that his illness was of slight importance. After a few days his friends became alarmed, and one more anxious and officious than the rest, warned his mother who was then with him, that unless better attended, her son would die, and so he did. His remains now repose in one of our beautiful cemeteries, and the day is not yet forgotten by many sad hearts when his body was borne through our streets and deposited in its final resting place. I shall never forget that surgeon for his heartlessness, nor forgive his neglect.

A Captain engaged in that murderous assault upon the Heights of Fredericksburg, had the misfortune to be struck in the thigh by a "minnie." He fell instantly, and rose as quickly to his feet, was assisted to the hospital, and the ball taken out by incision posteriorly. An amputation was decided upon. To this he demurred, saying that as he could stand and walk he knew the bone was not broken, and amputation unnecessary. Several surgeons examined and declared the bone shattered, for could they not feel with their fingers the sharp points? It was finally conceded to wait until a certain Surgeon, named by the Captain, could examine it, and if he coincided with them, he would yield and submit to an immediate amputation. He came, and in his examination had the satisfaction, after some exertion, to remove one of the splinters, and taking it to the light exclaimed, "By G—d, gentlemen, that man's thigh-bone is made of gutta-percha." Then examining his pants, it was found that the ball had struck the contents of his pocket, and carried the fragments of his gutta-percha comb into the wound, and it was these fragments that were mistaken for spicula of bone. I have seen the ball that did this mischief. The Captain's theory was better than that of the surgeon. He said, "if the bone is broken, how could I walk?" They answered, "if the bone is not broken, whence these fragments!" The limb was saved by accident, and is now as good as ever. The Captain has his own self will, and not the half dozen surgeons in attendance to thank that he now has two legs to walk with. It was perhaps a natural mistake, but if the Surgeons possessed as much good common sense as the Captain, it never should have occurred.

An assistant surgeon had his arm amputated in a hospital in Washington, and died in the night from hemorrhage. Was this not from ignorance or carelessness?

Another soldier had his arm amputated just above the elbow. The surgeon dressed it, applying fifteen yards of bandage, covered with oiled silk, and tied below and at the shoulder so tight as to ligate it, He also died. I could go on enumerating cases, but will only allude to the case of Col. Newman, as related by Dr. Swinburn of Albany, and published in some of the journals and New York papers. I would like to see the man that could read it and not feel indignant. For this class of Army Surgeons my remarks were intended; and from this class it would seem that Dr. Peters would "avert obloquy." I have never by word or thought "slurred" the careful, competent surgeons of the army. I know quite well how to appreciate their services. They need no defence, for I have not placed them on trial. As for the incompetents, (and I am sorry to say they are but too, numerous,) I shall never cease to condemn. Too many of their victims remain to mourn over their sufferings and their wrongs:

"It may be for years; it may be forever."

With these remarks I leave the Medical Army Staff to take a look at the Doctor's next paragraph. It is so long, contains so much, and proves so little, that I hardly know where to begin. The glibness and facility with which he disposes of the question at issue, can only be equalled by that of its invention and the mythical cases cited from a *very convenient* memory. He now says his "*cases*" all occurred within a short time, in one camp, and all attended with derangement of the digestive organs. Now I think the Doctor should begin to see through the mist and understand his "*cases*,' for he knows as well as I that the remedies used for the derangement of the digestive organs referred to, consisted of mercurials, saline aperients and aromatic sulphuric acid and opium. And from this treatment comes what? "Spontaneous salivation," of course. As he says he has consulted one of our oldest and most scientific physicians. I will also say for what it is worth, that some years ago I recollect being cautioned by an experienced physician and teacher, to be cautious in exhibiting calomel in connection with muriatic acid, for fear of salivation by the chemical change alluded to, and made so light of, by Dr. Peters. Whether the Doctor's adviser would have been benefitted by my counsel in his cases or not is of little consequence; of one thing I am fully convinced, and that is, *if he administered mercurials* to his patients. and they became salivated, he would not have been at a loss how to account for it. If Dr. Peters has not yet discovered the danger of administering acids and mercury, he has much to learn before I should be willing to trust myself to his care. And when he says with such apparent ecstacy to the profession: "There's a discovery for *you*," he had better say *for me*, for I can assure him it has occupied minds far superior to ours.

I have not said that acetic acid alone would change the mild chloride to the bi-chloride, but that some acids do, there is little doubt. The modus operandi would be as difficult to explain to his satisfaction as why a "Chimera ruminates in a vacuum." "No other paper has the news." This trifling expression is characteristic of one who having been blind, begins to see. I spoke of the recklessness of *some* army surgeons in the use of medicines and their manner of examination and prescribing. On this subject the Doctor is silent, and endeavors to demolish "Medicus" with his wit; but facts will be stubborn things, and he will find that time and experience will upset many a fine theory.

The Doctor seems to think very lightly of my quotation from Braithwaite, which I thought sufficient to demonstrate the object in view. It will not do to dodge from the real question to that of how *acetic acid* can change the chlorides. It must be remembered that we have often to rely upon others more experienced than ourselves for the facts to establish a single point; hence reference to those who have preceded us becomes indispensable. For the purpose of enlightening the minds of such as find their patients salivated after giving *small doses* of hydrargyrum, and are unable to account for it, because they gave only small doses, I will quote some cases:

"The use of six grs. of blue pill in divided doses, for three days, caused excessive salivation with great constitutional disturbance, offensive evacuations, copious sweating, bleeding from the nose, purple spots on the skin, dilated pupils, and such severe local disease that the teeth dropped out and the patient died in six days."—*Lancet, vol.* 1, *p.* 205.

"Three grs. corrosive sublimate given in three doses caused violent salivation and death."—*M. Colson in Arch. Gen. de Med. xii,* 84.

"Five grs. of blue pill given for three nights caused fatal salivation." *London Med. Gazette*, 1, 775.

"Two grs. calomel have caused ptyalism, extensive ulceration of the throat, exfoliation of the lower jaw and death."—*Trans. Dublin Coll. of Physicians, iv.* 91.

It is also known that three drachms of mercurial ointment have caused salivation and death. I would like to ask if soldiers ever use that article? Of course not, that Dr. Peters knows of, judging from his own statements. We expect of course these cases will be attributed to idiosyncrasies, which I am willing to grant.

Now I would like to ask Dr. P. one question, which may be very clear to his comprehension, but not so to mine. It is this: What are the idiosyncrasies I have quoted, and he has harped so much upon, caused by, if not by acids in the "*primæ viae*," causing chemical changes? Idiosyncrasies must be caused by something, and what more likely, than that mild mercurials are changed by some acid in the stomach, and hence the wonderful activity of the drug in the cases quoted? Then again it may not be known to Dr. P. that American calomel is often adulterated with corrosive sublimate. But where two grains of calomel cause severe salivation, I would like to ask the Doctor what constitutes the idiosyncrasy? What and where is it located? Unless he can explain I fear his wit and arguments will have about as much effect upon the medical, as the tail of a comet would upon the material world. If you explain, "*Posteritas sum hic res memor.*" If my statistics are wrong Dr. P. is alone to blame. The manner in which they are dodged is very amusing. I still think it strange that those six cases should occur in one month in the 21st, and not affect other regiments in the same proportion. When he said they occurred in the month of August, 1862, I knew that the story was apochryphal; and now they "certainly did occur, but it might be at some other time and place." Comment is unnecessary.

How ingeniously dates are disposed of. In his first paper he says that his "cases" occurred in the month of August, 1862. The facts stated in my former article in regard to the removal of the sick on the 9th of that month and the marching and fighting which followed left the whole statement in doubt; and if any further evidence were wanting, the manner in which he gets around it is *really convincing*. Hear him: "I do not know as precise dates are important, but as they have been alluded to, I will say that all these cases occurred in one camp, and previous to the 9th of August, 1862, some may have been seen in the latter part ofi July." First, it all happened in the month of August. Now he says it might be in July, and at some other camp, and certainly before August 9th. Why? Because when his cases were invented from a few cases of mercurial salivation he did not know that any one would question his correctness and his "literature" would pass unnoticed. According to his last statements his cases must have occurred at camp "Rufus King," which I have the best of reasons to doubt. Next, "Je ne sais oū le prendre." As to the "most effectual" gargle used, to wit, brandy and water, I deny that any brandy was used for any such purpose, and I know I am right when I assert that all used was to gargle well men's throats, and went to that "bourne" from whence good spirits "never return." I did not say that the disease was confined to "pregnant women and teething children." But that in men it was rare. As to the literature of the subject, I take no exceptions to his quotations, they are quite familiar to me. It is his own wonderful discoveries, cases, dates, and localities that I have intended to notice. It certainly was unfortunate that he gave dates so precisely in his first communication, and well perhaps that he was indefinite in his last statement as to time and place, for "Medicus" might have been *there* to see. I will cheerfully lend him my pen, if, "with the feather end dipt in acid," or *any thing* else he can obliterate his mistake. I have no doubt the surest method of obliterating it from the Doctor's memory, would be found in the free use of the "*most effectual*," certainly it is too palpable to be hid in the dense "cloud" of smoke attempted in his last communication.

With this subject I have done.

Truly yours, MEDICUS.

